# Burden and factors for the early resumption of sexual intercourse in the puerperium among new mothers at Kawempe national referral hospital and Mengo hospital, Uganda

**DOI:** 10.4314/ahs.v23i4.45

**Published:** 2023-12

**Authors:** Edith Namulema, Sarah Nakubulwa, Lubega Muhamadi

**Affiliations:** 1 Department of Public Health and Infectious Diseases, Mengo Hospital, P.O. Box 7161, Kampala, Uganda; 2 Department of Obstetrics and Gynaecology, Makerere University, College of Health Sciences P.O.Box 7072 Kampala, Uganda; 3 Lubega Institute of Nursing and Health professionals, Iganga, Uganda

**Keywords:** Early resumption, Sexual intercourse, postpartum mothers, determinants

## Abstract

**Background:**

Early resumption of sexual intercourse in the puerperium is a concern for couples because it is often not discussed during pre-natal or postpartum care.

**Objective:**

This cross-sectional survey aimed to establish the current burden and factors associated with the early resumption of sexual intercourse within the puerperium at the National Referral Hospital and Mengo Hospital.

**Methods:**

We conducted a descriptive cross-sectional study among 445 parous women attending the six-week postpartum review and the young child clinic at Kawempe National Referral and Mengo Hospitals between March and May 2021.

**Results:**

The prevalence of ERSP within the puerperium was 39%. This study's earliest time to resume sexual intercourse was one week; the majority had resumed by week four (9.2%). Factors associated with the early resumption of sexual relations were the person's tribe, going to the husband's home after birth, and parity. The prevalence of sexual morbidities was 13%. Seventy-five (75%) of mothers did not receive information from the health care workers on when they can resume sex following childbirth.

**Conclusion:**

Puerperal sexual intercourse is still prevalent in Uganda. Interventions to reduce the resumption of sexual intercourse in the puerperium should focus on these determinants to delay puerperal sexual intercourse.

## Introduction

Early resumption of sexual intercourse, defined as any vaginal intercourse occurring within six weeks or earlier after delivery, is a concern for couples. However, it is often not discussed during pre-natal or postpartum care. 1, 2 The postpartum period profoundly impacts a woman's quality of sexual life as the couple adapts to the new addition to their family and alterations in their sexual functioning. 3 Sexual functioning in the postpartum period is a concern for women because many are unaware of the changes to their sexual health after childbirth. 1 Therefore, the resumption of sexual activities will depend on several variables, such as the mother's general health, her relationship with the partner, her emotional readiness, and her adaptation to the new maternal role. 4

Globally, the resumption of vaginal sexual intercourse for couples early in the postpartum period continues to be high. Studies show that postpartum sexual abstinence ends before the six weeks following childbirth. 5-9 Early resumption of sexual intercourse contradicts the World Health Organization (WHO) guidance, which recommends that women who have undergone childbirth abstain from sex for six weeks to allow the reproductive tract to return to the non-pregnant state. 10 The guidance is because the resumption of sexual intercourse early in the postpartum endangers the mother's health and is associated with sexual morbidities, which may significantly affect the mother's physical, psychological, and social wellbeing. 11

In Uganda, studies show that the prevalence of ERSP ranges between 21 to 58%. 5, 12, 13. Women who deliver from health units are allowed home within hours of childbirth without information on when and where to obtain additional care, including sexual health. 19 In addition, the women's negotiating power regarding sex with their partners is limited and may force women to resume sexual intercourse early. 14 However, the gaping episiotomy wounds resulting from the early resumption of sexual intercourse in the puerperium are associated with sexual dysfunctions and psychosocial problems such as loss of libido and depression. 15 Therefore, this cross-sectional survey aims to establish the current burden and factors associated with early resumption of sexual intercourse within the puerperium (ERSP) among new mothers in Kawempe National Referral (Kawempe NR) and Mengo Hospitals.

## Methods

We conducted a descriptive cross-sectional study among women who attended the six-week postpartum review clinic and the young child clinic at Kawempe NR and Mengo Hospitals in Kampala, Uganda. Between March and May 2021, we interviewed 445 mothers in a ratio of 1:10, 45 at Mengo Hospital, and 400 at Kawempe NR Hospital. Two female research assistants administered the structured questionnaire and obtained written informed consent from the participants. We excluded those who refused to consent. To ensure total confidentiality, we conducted the interviews with each participant in a private room. We divided the questionnaire into three sections: demographics; (age, occupation, parity, and mothers; address upon childbirth); Obstetrics history (parity, mode of last delivery, episiotomy/perineal injury, and puerperal complications); the resumption of vaginal sexual intercourse, and any sexual morbidities resulting from ERSP. The questionnaires were entered directly into Open Data Kit (ODK) on Google drive and checked for errors. We analysed the data using Stata Version 14.0. This study received approval from the ethics and research committee of Mengo Hospital Research and Ethics Committee (45/08-2020) and the Uganda National Council for Science and Technology (HS1050ES).

## Results

Data analysis showed that of the 445 mothers who participated in the study, (37%) were young mothers aged less than 25 years. Fifty four percent either had no education or ad attained primary level education. Almost half of the mothers were married (46.2%), The mean parity was 3.5 (S.D. 1.6). More than half of the women were from the Baganda tribe (55%), and 34% were unemployed. On the other hand, most spouses were privately employed (48%), with only 5% unemployed.

About 75% (339/445) of mothers did not receive information from the health care workers on when they can resume sex following childbirth. Instead, majority 154/430 (35.81%) sought this information from their husbands, while more than one third of the women 159/430 (36.98%). did not ask anyone. Among those that received health education, the most common timing prescribed by healthcare workers was six weeks to two months. This duration was prescribed to about 42% of new mothers (n=45 of the 106). Health workers told Eleven mothers (11/106) to resume sex in less than a month, while the 9% (9/106) were told to resume sex after six months.

About 77% (n=339 of 445) went to the husband's home after delivery and were protected from early sex. Thirteen, 13% (n = 56 out of 445) of new mothers reported coercion into an early resumption of sexual intercourse, of whom 13% (n=23 of 175) experienced sexual morbidities.

Approximately 67.6% of the women reported that their sexual desires did not take priority over the men, likely resulting in early sex.

**Figure 1:**
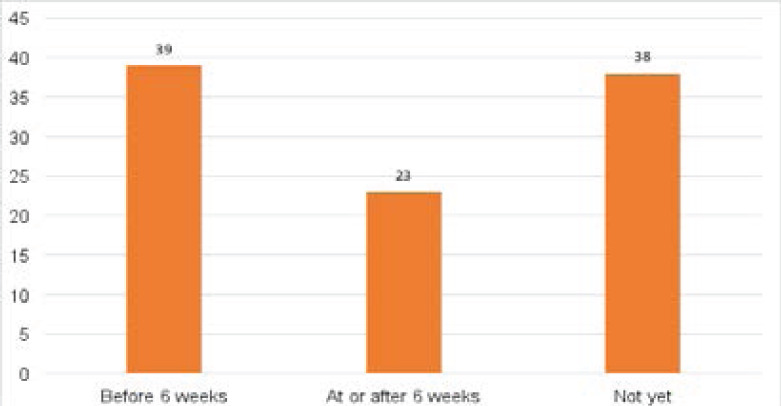
Prevalence of early resumption of sexual intercourse within the puerperium (ERSP) among new mothers by and before six weeks

Figure 1 above shows that the prevalence of ERSP before six weeks (i.e., within the puerperium) was 39% (n=175 of 445), and prevalence at or after six weeks was 23% (n=101 of 445 new mothers), about 38% (n=169 0f 445) of new mothers had not resumed sexual intercourse.

**Figure 2:**
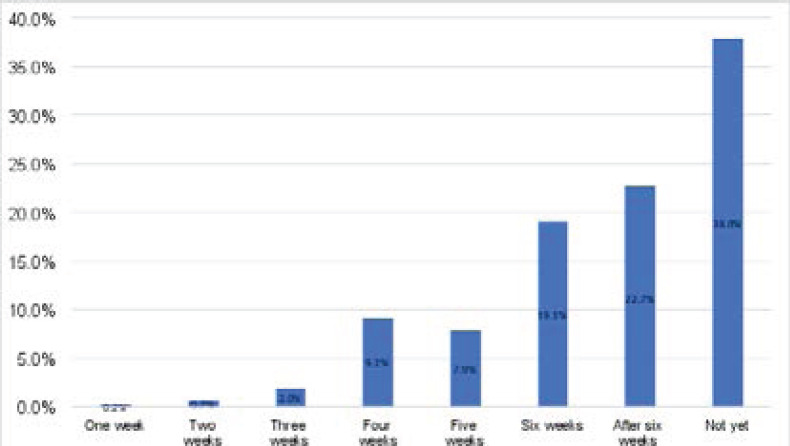
Percentage of early resumption of sexual intercourse within the puerperium (ERSP) among new mothers

Figure 2 above shows that this study's earliest time to resume sexual intercourse was one week with one individual (0.2%). Sixteen (9.2%) resumed at four weeks, followed by fourteen (7.9%) at five weeks. More that more than one third of the new mothers had not yet resumed sexual intercourse at the time of the study (38%).

Table 2 below show the bi variate analysis using Stata 14, the social demographic factors that differed between new mothers who resumed sexual intercourse before six weeks and those who resumed sexual intercourse at or after six weeks. The mothers' age p<0.03, tribe p< 0.02, mode of delivery p<0.05, occupation p<0.01, parity <0.01, mothers who returned to husband's home after delivery (p-values < 0.01) and spouses' occupation p<0.02 were associated with ERSP.

**Table 2:** Bivariate analysis of the factors associated with the early resumption of sexual intercourse in the puerperium. (n=445)

Factor		Resume before six weeks	Resume at or after six weeks	p-value
		N = 175 39%	N = 270 61%	
Age*(in years)	Less than 25	58 (33.1%)	105 (38.9%)	0.03
	25-30	51 (29.1%)	95 (35.2%)	
	31-35	34 (19.4%)	45 (16.7%)	
	36 and older	32 (18.3%)	25 (9.3%)	
				
Education	Tertiary	35 (21.0%)	55 (20.8%)	0.31
	Secondary	49 (29.3%)	61 (23.1%)	
	None of primary	83 (49.7%)	148 (56.1%)	
				
Tribe*	Muganda	82 (46.9%)	161 (59.6%)	0.02
	Musoga	25 (14.3%)	35 (13.0%)	
	Other tribes*	68 (38.9%)	74 (27.4%)	
				
Religion	Catholic	37 (22.2%)	72 (27.6%)	0.32
	Anglican	75 (44.9%)	95 (36.4%)	
	Moslem	36 (21.6%)	65 (24.9%)	
	Other religion	19 (11.4%)	29 (11.1%)	
				
Spouse Occupation*	Government	30 (17.3%)	32 (11.9%)	0.02
	Private	91 (52.6%)	123 (45.7%)	
	Self-employed	52 (30.1%)	109 (40.5%)	
	Not employed	0 (0.0%)	5 (1.9%)	
				
Birth Mode	SVD	117 (67.2%)	185 (68.8%)	0.05*
	Operative	44 (25.3%)	77 (28.6%)	
	Assisted	13 (7.5%)	7 (2.6%)	
				
Go to husband's home after birth*	Yes	153 (88.4%)	186 (69.1%)	<0.01
	No	20 (11.6%)	83 (30.9%)	
				
Sex Education from HCW	Yes	47 (27.2%)	59 (21.9%)	0.20
	No	126 (72.8%)	211 (78.1%)	
				
New Mother Occupation*	Government	15 (8.6%)	20 (7.4%)	<0.01
	Private	57 (32.8%)	52 (19.3%)	
	Self-employed	45 (25.9%)	104 (38.7%)	
	Not employed	57 (32.8%)	93 (34.6%)	
				
Parity, *mean (S.D.)* *		3.8 (1.6)	3.3 (1.5)	<0.01
				
Contraceptive use	Yes	56 (32.2%)	101 (37.5%)	0.25
	No	118 (67.8%)	168 (62.5%)	
				
Marital Status	Married	88 (50.6%)	117 (43.3%)	0.14
	Other	86 (49.4%)	153 (56.7%)	

HCW: Health Care Worker

* P-values < 0.05; indicating variables that are associated with ERSP

Resuming sexual intercourse at or after six weeks includes new mothers who have not yet resumed sexual relations

After adjusting for the significant variables, Table 3 above shows that statistically significant factors associated with early resumption of sexual relations were the person's tribe, going to the husband's home after birth, and parity. New mothers who did not go to the husband's home after delivery were 69% less likely (OR = 0.31, 95% CI = 0.17 – 0.54) to resume sexual relations before six weeks than those who went to the husband's home after delivery. Higher parity was positively associated with the early resumption of sexual relations. New mothers with a higher parity were 23% (0R = 1.23; 95 CI% = 1.03 – 1.46) more likely to resume sexual relations before six weeks than those with a lower parity. Compared to those belonging to the Muganda tribe, new mothers belonging to other tribes were 84% (OR= 1.84 95% CI 1.1.7-2.92) more likely to resume sexual intercourse within the puer-perium.

**Table 3:** Multivariate analysis of the factors associated with resuming sexual relations before six weeks among new mothers in Kawempe NR and Mengo Hospital

Factors	Odds Ratio	95% Confidence interval		P-value
**Age (in years)**				
Less than 25	Reference	Lower CI	Upper CI	
25-30	0.68	0.37	1.21	0.19
31-35	0.79	0.39	1.62	0.52
36 and older	1.03	0.42	2.51	0.96
**Tribe**				
Muganda	Reference			
Musoga	1.184	0.63	2.21	0.60
Other tribes*	1.846	1.17	2.92	0.01
				
**Spouse Occupation**				
Government	Reference			
Private	1.04	0.56	1.95	0.90
Self-employed	0.69	0.34	1.40	0.30
Not employed	*Only five people in this group, not enough for analysis			
				
Go to husband's home after birth*	0.31	0.17	0.54	<0.01
				
**New mother occupation**				
Government	Reference			
Private	1.65	0.73	3.73	0.23
Self-employed	0.66	0.28	1.53	0.33
Not employed	0.91	0.38	2.18	0.83
				
Parity*	1.23	1.03	1.46	0.02

## Discussion

The present study indicates that the early resumption of sexual intercourse for new mothers continues to be high. In this study, the prevalence of ERSP was 39%, a finding similar elsewhere in Nigeria, Turkey, and Australia. 6, 7, 16 This finding could be a function of information gaps for the mothers since 75% were not educated on the resumption of sexual intercourse after childbirth by the health workers.

The prevalence of ERSP in this study was higher than in a prior study in Uganda 5, and South-eastern Nigeria 17, but much lower in other studies in Ethiopia and England.^7^, ^8^, ^19^. The decline in ERSP noted in this study could result from attaining higher basic education levels among our women. For example, 46% had attained secondary and tertiary education in this study and were empowered to decline sexual requests from their partners. Furthermore, traditional and cultural practices favouring early resumption of sexual intercourse may not be strongly observed by the Baganda women, who were the majority of participants. Equally, the differences in ERSP prevalence could be due to differences in beliefs and practices around puerperal sexual health in these mothers. 8

Socio-demographic and behavioural factors were the main determinants for the resumption of postpartum sexual activity. The multivariate analysis's most significant predictors of the ERSP in this study were a person's tribe, going to the husband's home after birth, and parity.

New mothers from other tribes besides the Muganda tribe were 84% (AOR= 1.84 95% CI 1.1.7-2.92) more likely to resume sexual relations before six weeks. The association between one's tribe and ERSP is similar to a prior Ugandan study on tribal customs and puerperal sexual intercourse. 11 Indeed some cultures in Uganda believed that a woman was expected to resume sexual intercourse within the first week after delivery “to bring good health to the baby.” 11 Besides, amongst the Banyankole tribe in Uganda, spouses are culturally obliged to resume sexual intercourse shortly after delivery to celebrate the new baby's arrival. 19 Hence, ERSP in this study, could be a function of traditional values, beliefs, and norms, and should not be underestimated. These values can affect the family, the community, and society, and may play an essential part in shaping people's sexual lives.

A higher parity was positively associated with the early resumption of sexual intercourse. New mothers with a higher parity were 23% (AOR = 1.23; 95 CI% = 1.03 – 1.46) more likely to resume sexual relations before six weeks than those with a lower parity. The association between higher parity and ERSP contrasts studies in Uganda, where women with fewer children resumed sexual intercourse in the puerperium.^5^ Indeed, women with higher parity are more likely to be experienced and to assess healing and wellbeing better.

Spousal pressure and fear that their man might go for extra-marital relations were reasons for woman's return to their husbands' homes soon after childbirth. Going to the husband's home after birth was significantly associated with ERSP and therefore exposed mothers to an early resumption of sexual intercourse. New mothers who did not go to the husband's home after delivery were 69% less likely (AOR = 0.31, 95% CI = 0.17 – 0.54) to return to early sex. Returning to the marital home after delivery and its association with ERSP has been reported in similar to studies elsewhere in Nigeria and Malawi, where proximity to the partner led to sexual demand of the partner and, subsequently, ERSP. 20-22 Therefore, the cultural practice of new mothers staying with their in-laws or at their parents' homes after childbirth is one-way culture can prohibit early resumption of sexual intercourse after childbirth. 23

The time to resume sex within the puerperium varies considerably in literature. In this study, the meantime for resumption of sexual intercourse was four weeks + 2 weeks, similar to a study in Nigeria 24 but a much shorter duration than in a similar setting in Uganda and Nigeria. 11, 25 Our study demonstrated a decline in women resuming sex in the first week following childbirth at 0.2% compared to a similar study in Uganda at 8.5% within the first week during the puerperium. 11 At two weeks, a smaller percentage (0.7%) resumed intercourse than in Nigeria, where 15.4% resumed within two weeks of delivery.^25^ In the first two weeks of childbirth, the ERSP could be a function of coercion, 26 intimate partner abuse, 27 lack of information, 28 or the lack of social support from the partners. 29

By four weeks, 9.2% of mothers had resumed sexual intercourse. Resuming sexual intercourse by the fourth week was reported in a similar to study among American women 30. Furthermore, a much smaller percentage of women (27%) in this study had resumed sexual intercourse by the fifth and sixth weeks postpartum compared to 41.2% in Nigeria. 25 The decreases in ERSP at the different intervals during the puerperium could be that participants in this study were young, with fewer children, and could have preferred to wait longer due to the lack of knowledge or the fear of getting hurt. Also, the younger respondents could be unmarried women who lived with their parents soon after childbirth.

Only 25% of women received information from health workers on when to resume sexual intercourse following childbirth. The lack of communication on postpartum sexual health is reported elsewhere in Canada and the USA, where sexual health discussions were not conducted. 28,30 The lack of prioritization of sexual health education talks could be a function of knowledge gaps, stigma, and lack of time due to the long queues in the MCH clinics. Therefore, health workers must consider puerperal sexual health as a critical component to address during the Ante Natal and Post Natal clinics to avoid ERSP.

In this study, half of the men sought information regarding the resumption of sexual intercourse from friends. In contrast, more than one-third of the women sought the same information from their spouses (35.81%), and another one-third did not consult anyone (36.98%). The failure to seek information on resumption of sexual intercourse contrasts study results in Iran, where women may find it easier to share their problems with friends. 31 Therefore, questions on sex should be asked routinely in medical practice by the providers.

Thirteen percent (13%) of mothers in this study reported sexual coercion. Although sexual coercion was lower than in a previous study in Uganda, 11 sexual coercion during pregnancy is reported elsewhere in Ethiopia and Iran. 32, 33 Furthermore, reports indicate that some mothers requested not to be discharged quickly from the hospital following childbirth, fearing sexual coercion by their partners before they heal. 5 Additionally, women return to the hospital with gaping episiotomies in the postnatal period, indicating they had sexual intercourse before the perineum entirely healed. 15 Sexual coercion could be a function of male partners' knowledge gaps on intimate partner violence. Therefore, coercion remains a factor predisposing mothers to early sex soon after birth and subsequent morbidity. Strategies to assess sexual coercion and knowledge gaps must be addressed in the MCH clinics.

In this study, the prevalence of sexual morbidities among new mothers was low, 13% (n=23 of 175), contrasting other studies in Australia and Nigeria. 34, 35 The low percentage in our study could be that mothers offered favourable answers since sex discussion remains taboo in this patient population. 14

Finally, the use of contraception p=0.25, religion p=0.32, and marital status p=0.14 was not associated with ERSP. The finding on contraceptive use and its lack of association with ERSP was reported elsewhere in Nigeria. 20 Mothers who used contraception were more likely to be confident and empowered about their sexual health, more aware of the consequences of ERSP, and ready to address them. Similarly, unlike other studies on Muslim women, the Muslim religion did not influence the early resumption of sexual relationships within the puerperium as reported elsewhere in Cote d'Ivoire and Iran. 36, 37 Thirty-six 36 (21.6) Muslim women resumed sex before six weeks in this study. The early resumption of sexual intercourse among Muslim women is contrary to Islamic teachings, which prohibits sexual intercourse as long as postpartum bleeding is present (i.e., usually 40 days after childbirth). 37 Hence, ERSP among this cohort of Muslim women could be a function of knowledge gaps and sub-missiveness. Submissiveness to sexual demand may depend on several factors, such as dowry payments which may lead the woman to obligations to please her husband sexually, including the puerperium. 14

## Conclusion

Early resumption of sexual intercourse in the puerperium is still prevalent in Uganda. A person's tribe/culture, going to the husband's home after birth, and multi-parity were determinants for puerperal sex. This study shows the need for interventions and education on when to resume sexual intercourse following childbirth during the maternal and child health clinics.

## Limitations

Selection bias at the study clinics restricted the generalization of the study findings to rural dwellers. Being urban health centers, rural-dwelling women, likely to be of low literacy and socioeconomic status, were not represented in this study. Sexual health discussions are still taboo in the study setting, especially in the postpartum period, when women are expected to abstain and concentrate on the baby's care. Therefore, socially desirable responses may have been given concerning ERSP.

## Figures and Tables

**Table 1:** Distribution of demographic variables among new mothers (n=445)

New Mother Characteristics		N (%)
		N = 445
Age (in years)	Less than 25	163 (36.6%)
	25-30	146 (32.8%)
	31-35	79 (17.8%)
	36 and older	57 (12.8%)

Education	Tertiary	90 (20.9%)
	Secondary	110 (25.5%)
	None of primary level	231 (53.6%)

Tribe	Muganda	243 (54.6%)
	Musoga	60 (13.5%)
	Other tribes*	142 (31.9%)

Facility	Mengo Hospital	120 (32.4%)
	Kawempe Hospital	251 (67.6%)

Spouse Occupation	Government	62 (14.0%)
	Private	214 (48.4%)
	Self-employed	161 (36.4%)
	Not employed	5 (1.1%)

Parity, mean (SD) 3.5 (1.6)		

New mother Occupation	Government	35 (7.9%)
	Private	109 (24.6%)
	Self-employed	149 (33.6%)
	Not employed	150 (33.9%)

Marital Status	Married	205 (46.2%)
	Other status*	239 (53.8%)

Other tribes included: Banyankole, Bakiga, Basoga, Banyarwanda, Banyoro
